# Fish Protein Hydrolysate from Sulfated Polysaccharides Extraction Residue of Tuna Processing By‐Products with Bioactive and Functional Properties

**DOI:** 10.1002/gch2.202200214

**Published:** 2023-01-29

**Authors:** Shahab Naghdi, Masoud Rezaei, Mehdi Tabarsa, Mehdi Abdollahi

**Affiliations:** ^1^ Department of Seafood Processing Faculty of Marine Sciences Tarbiat Modares University P.O. Box 46414‐356 Noor Iran; ^2^ Department of Life Sciences–Food and Nutrition Science Chalmers University of Technology Gothenburg SE 412 96 Sweden

**Keywords:** ACE‐inhibitory activity, biorefineries, fish by‐products, functional properties, protein hydrolysates

## Abstract

The ethanol‐induced precipitation after enzymatic hydrolysis commonly used for sulfated polysaccharide extraction from marine resources wastes a large amount of proteins. Here, possible extraction of fish protein hydrolysates (FPH) from the ethanol residue of sulfated polysaccharide precipitation from head, bone, and skin of skipjack tuna is investigated. Antioxidant, antibacterial, angiotensin I‐converting enzyme (ACE) inhibitory activities and functional properties of the recovered FPHs are also evaluated. A degree of hydrolysis of 40.93, 38.13, and 37.23 is achieved for FPH from head, bone, and skin, respectively. FPH from the head presents the highest antioxidant and ACE inhibitory activity as well as foam/emulsion capacity among all the FPHs. The FPHs are all able to inhibit three Gram‐positive bacteria and three Gram‐negative bacteria to varying degrees and have a water solubility >65%. Altogether, the results demonstrate great potential for recovery of bioactive/functional peptides from the residue of sulfated polysaccharide extraction process enabling efficient biorefining of aquatic resources.

## Graphical abstract

This study proves that the ethanol residue remaining during polysaccharide extraction from skipjack tuna by‐products can be a source for parallel extraction of protein hydrolysates with good bioactivity and functional properties and can be employed as a natural source of antioxidant and antibacterial compounds that can be used in the food and nutraceutical industries.

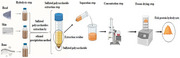

## 1 Introduction

In recent decades, aquaculture and seafood processing has been receiving great attention from industry and researchers to support the protein demands of an increasing human population.^[^ ^1^, ^2^ ^]^ Consequently, a massive amount of by‐products are generated after seafood processing, including heads, tails, fins, spines, viscera, and skin, which are responsible for 75% of the total weight of fish.^[^ ^1^, ^2^ ^]^ However, these by‐products are a great source to extract bioactive compounds including protein hydrolysates, sulfated polysaccharide, EPA, and DHA with numerous bioactive properties such as antioxidant, antibacterial, antihypertensive, angiotensin I‐converting enzyme inhibitory.^[^ ^3^ ^]^

To extract these natural products from fish by‐products, many strategies have been developed in recent years, such as enzyme‐assisted extraction, ultrasonic‐assisted extraction, microwave‐assisted extraction, accelerated solvent extraction, pressurized water extraction, and ultrahigh‐pressure extraction.^[^ ^1^, ^2^ ^]^ For instance, the first goal of an enzymatic hydrolysis method is to obtain high‐quality protein fragments called hydrolysate with functional properties, but enzymatic hydrolysis under optimized conditions can generate protein hydrolysates with bioactive properties.^[^ ^3^ ^]^ However, more recently the enzymatic hydrolysis process has been used for the destruction of connections between protein‐polysaccharides for extraction of sulfated polysaccharides from fish by‐products. This initial enzymatic hydrolysis step is then followed with the ethanol or cetylpyridinium chloride (CPC) induced precipitation to recover the extracted polysaccharides as precipitate.^[^ ^4^ ^]^ Consequently, after polysaccharides precipitation which normally encompasses a very small portion of marine biomass, a huge volume of ethanol or CPC remains and is discarded while it still contains hydrolyzed proteins that could be recovered from the ethanol phase. This will provide a unique opportunity for parallel extraction of polysaccharides and protein hydrolysate from fish products. Co‐extraction of these two products can increase the overall profitability of the process by increasing product diversity and revenue. It also enables more efficient utilization of marine resources by taking care of side streams. In this context, Abdollahi et al.^[^ ^5^ ^]^ developed a biorefinery concept for sequential recovery of collagen and collagen hydrolysate from the sediment residue created during pH‐shift‐based protein isolation from silver carp (*Hypophthalmichthys molitrix*). However, as far as we know, parallel extraction of protein hydrolysates from waste streams generated during the extraction of sulfated polysaccharides from fish processing by‐products has not been documented.

In addition, depending on their origin and extraction process, fish protein hydrolysates (FPH) with various nutritional properties related to their amino acid content and sequence will be generated.^[^ ^3^ ^]^ These two factors will also affect the possible bioactive properties of the recovered protein hydrolysates. Antioxidant, antibacterial, and angiotensin I‐converting enzyme (ACE) inhibitory peptides have been reported previously for protein hydrolysates from various fish byproducts.^[^ ^1^, ^2^ ^]^ In addition, hydrolyzed peptide due to their hydrophobicity and charge have been reported to show multifunctional activities based on their structure.^[^ ^1^, ^2^ ^]^ There is little information available on how the type of by‐products (head, bone, and skin) and ethanol precipitation can impact the bioactive properties of their protein hydrolysates that have been targeted in this study.

Thus, the purpose of this study was to i) investigate the possibility of recovering FPH from ethanol residues remaining after precipitation of sulfated polysaccharides from skipjack tuna (*Katsuwonus pelamis*) by‐products and ii) assess how by‐products including head, bone, and skin affect bioactive properties (antioxidant, antibacterial, ACE inhibitory), and functional properties (emulsion and foaming activity).

## 2 Result and Discussion

### 2.1 Degree of Hydrolysis (DH)

As can be seen in **Table** **1**, the highest DH (29.79 ± 1.27%) was observed in FPH‐head which was significantly different from the two other FPHs but no significant difference was seen between FPH‐bone (28.32 ± 0.88%) and FPH‐skin (27.77 ± 0.56%). The higher DH obtained for the three FPHs could be described by a long time of hydrolysis process used here, which was about 12 h.^[^ ^6^, ^9^ ^]^ The higher DH obtained for head could be related to the higher ratio of muscle tissue to collagenous residue (bone and skin) in the head compared with head and skin which are typically more difficult to be hydrolyzed to smaller peptides. Enzymes cleave less compact parent proteins more quickly than compact core proteins when they interact with insoluble proteins, breaking loosely bound polypeptide chains.^[^ ^9^ ^]^ It is well known that the protein substrate, enzymes used, the conditions, and DH have a major impact on various physicochemical and biological properties of protein hydrolysates.^[^ ^10^ ^]^

**Table 1:** Degree of hydrolysis of fish protein hydrolysates (FPH) recovered from ethanol residue of tuna head, bone, and skin

Samples	FPH‐head	FPH‐bone	FPH‐skin
Degree of hydrolysis [%]	29.79 ± 1.27 ^a^	28.32 ± 0.88 ^b^	27.77 ± 0.56 ^b^

Values are mean of triplicate determinations ± SD

Small letter in each row shows statistically significant differences.

### 2.2 Amino Acid Composition of Recovered FPHs

Amino acid composition and levels are key characteristics that determine the function of peptides.^[^ ^11^ ^]^ To clarify the probable influence of the amino acid profile on the functional, antioxidant, and antibacterial activity of the recovered FPHs, the amino acid profiles of recovered FPHs are shown in **Table** **2**. As can be observed, the contents of essential amino acids in FPH‐head, FPH‐bone, and FPH‐skin were 46.41, 44.89, and 33.47 (g/100 g protein), respectively, which were comparable to an earlier report by Noman et al.,^[^ ^12^ ^]^ who used alcalase and papain to produce protein hydrolysate from Chinese sturgeon (*Acipenser sinensis*) meat and their by‐products. Although, the essential amino acid content in our recovered hydrolysates was lower than FPH obtained via alcalase (49.62 g/100 g protein) by Noman et al.^[^ ^12^ ^]^ The content of the essential amino acids such as lysine (Lys) and leucine (Leu) was higher than 5 g/100 g protein in FPH‐head, and FPH‐bone, while it was lower than 5 g/100 g protein in FPH‐skin, also the highest content of Lys and Leu were seen in FPH‐bone. It was reported by Zou et al.^[^ ^13^ ^]^ that peptides that contained these amino acids, tyrosine, histidine, methionine, and lysine, showed strong radical scavenging ability in the oxidation process, so much so that their content in the recovered hydrolysates in the present work ranked as follows: FPH‐skin> FPH‐head> and FPH‐bone. Alternatively, hydrophobic amino acids such as glycine, alanine, methionine, valine, phenylalanine, isoleucine, and proline can scavenge radicals on membrane lipid bilayers.^[^ ^13^ ^]^ Table 2 shows that FPH‐head has the highest amount of hydrophobic amino acids. Besides, histidine due to the protonation of the imidazole ring can act as a hydrogen donor, and improve antioxidant capacity, which FPH‐skin showed the highest amount of histidine.^[^ ^14^ ^]^ Interestingly, aromatic amino acids such as tyrosine, phenylalanine, and tryptophan have notable antioxidant activity and a powerful chelating effect, and also they can chelate Fe^2+^ and Cu^2+^, thereby diminishing their activity, and can hamper lipid peroxidation,^[^ ^15^ ^]^ which in the present work FPH‐bone shows the highest content of those amino acids. The existence of excess electrons in glutamic acid and aspartic acid, which can contribute to antioxidant properties, was reported by Park et al.^[^ ^16^ ^]^ In the present research, the highest contents of those amino acids were seen in FPH‐head hydrolysate sample. Based on previous studies, peptides with high content of amino acids including arginine, alanine, valine, leucin, and lysine were introduced to having antibacterial properties, and peptides containing cationic amino acids demonstrated the greatest antibacterial activity against pathogenic bacteria as well.^[^ ^11^, ^17^ ^]^

**Table 2:** Total amino acid contents (g/100 g) of fish protein hydrolysates (FPH) recovered from ethanol residue of tuna head, bone, and skin

Amino acid	FPH‐head	FPH‐bone	FPH‐skin
Aspartic acid	11.32	10.33	5.49
Glutamic acid	12.24	16.43	11.03
Serine	3.64	3.22	2.72
Histidine	2.37	2.55	8.63
Glycine	5.01	6.37	17.83
Threonine	4.06	3.86	3.82
Arginine	3.97	6.13	7.49
Alanine	5.32	6.31	17.63
Tyrosine	3.16	2.12	4.13
Methionine	3.27	2.57	1.63
Valine	5.71	6.31	2.41
Phenylalanine	5.31	4.26	2.16
Isoleucine	7.37	5.37	1.42
Leucine	6.93	7.24	3.83
Lysine	7.31	10.27	3.73
Cysteine	0.92	0.34	1.71
Proline	12.09	6.32	9.83
Essential amino acid	46.41	44.89	33.47
Hydrophobic amino acid	51.01	44.75	56.74
Aromatic amino acids	8.47	6.38	6.29

### 2.3 SDS‐PAGE Analysis

**Figure 1:**
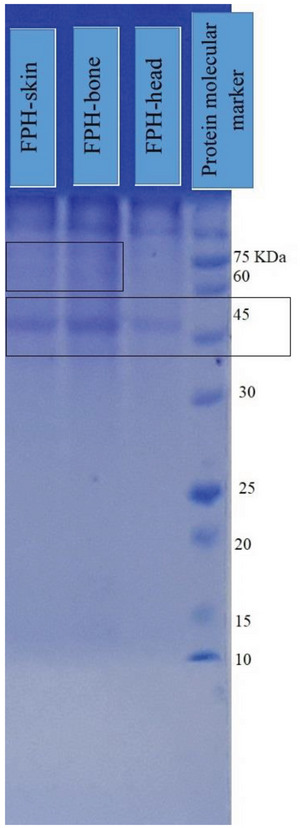
SDS‐PAGE image of fish protein hydrolysates (FPH) recovered from ethanol residue of tuna head, bone, and skin.

SDS‐PAGE is a helpful tool to study the breakdown and hydrolysis of proteins and peptides. It is also used to investigate and compare the efficiency of different enzymes in the same or different conditions.^[^ ^18^ ^]^ SDS‐PAGE determined the molecular weight of recovered FPHs (**Figure** **1**). The recovered FPHs showed a band in the range of ≈ 45 kDa for FPH‐head, FPH‐skin, and FPH‐bone. Also, FPH‐bone and FPH‐skin had weak bands in the range of 65–75 kDa in which no bond was seen for the FPH‐head. These results could be related to the DH for samples in which FPH‐head displayed the highest DH. According to previous studies, a varying degree of protein breakdown and the duration of hydrolysis could result in different SDS‐PAGE profiles of recovered FPHs.^[^ ^18^, ^19^ ^]^ The protein hydrolysates of the Taiwan mackerel (*Scomber australasicus*) steaming juice (MSJ) were found to have different bands of different molecular weights between 28 and 180 kDa in SDS‐Page analysis by Panjaitan et al.,^[^ ^20^ ^]^ in which the protein was mostly above 63 kDa in defatted MSJ, while mackerel muscle proteins were present in bold bands around 35, 48, and 100 kDa. Qara and Habibi,^[^ ^19^ ^]^ reported that hydrolysates obtained by Alcalase and Neutrase from Kilka (*Clupeonella cultriventris Caspian*) indicated higher levels of peptides with MW below 30 kDa. In contrast, Pepsin and Protamex produced hydrolysates that predominantly contained peptides of greater than 30 kDa, as found in the present study. Furthermore, previous reports indicated that bioactive peptides have molecular weights of less than 50 kDa, and are normally composed of 3–20 essential amino acids.^[^ ^21^ ^]^

### 2.4 Antioxidant Properties

#### 2.4.1 DPPH^·^ Radical Scavenging Activity

**Figure 2:**
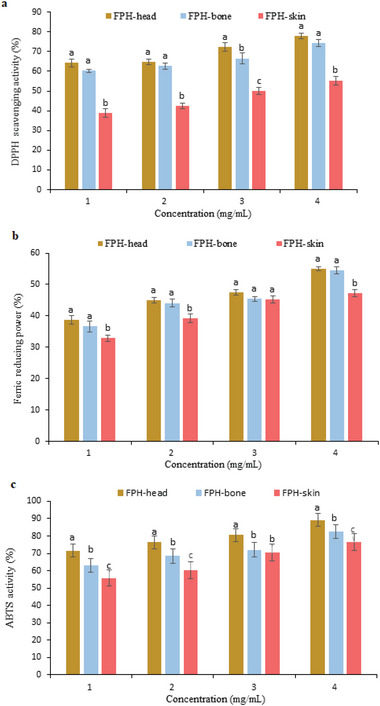
a) DPPH radical scavenging activities, b) Iron chelating activity, and c) ABTS radical scavenging activity of fish protein hydrolysates (FPH) recovered from ethanol residue of tuna head, bone, and skin.

In order to measure the antioxidant capacity of bioactive compounds, DPPH scavenging has been used, which is considered an easy and rapid method.^[^ ^22^ ^]^ DPPH scavenging activity of recovered fish protein hydrolysates (FPH‐head, FPH‐bone, and FPH‐skin) are shown in **Figure** **2**a. In all FPHs, an increasingly dose‐dependent DPPH scavenging activity trend was seen. FPH‐head showed a higher DPPH scavenging activity than FPH‐bone and FPH‐skin that had no significant difference with FPH‐bone except in concentration of 3 mg mL^−1^, but it showed a significant difference with FPH‐skin. The higher degree of hydrolysis data earlier documented in Table 1 for FPH‐head, could be a reasonable link to this result. This result was in agreement with the reported results by Foh et al.^[^ ^22^ ^]^ who have used three enzymes including Alcalase 2.4 L, Neutrase, and Flavourzyme to hydrolyze tilapia (*Oreochromis niloticus*) minced meat, and they stated that the hydrolysates with the higher degree of hydrolysis showed better DPPH scavenging activity. Additionally, Rios‐Herrera et al.^[^ ^1^ ^]^ showed that DPPH activity was increased by increased hydrolysis of catfish *Bagre panamensis* muscle hydrolysates, and that DH was associated with DPPH radical scavenging activity in a significant, positive and favorable manner (*P* < 0.001). The presence of low‐molecular‐weight peptides in hydrolyzed proteins is associated with a significant increase in antioxidant activity, which is well known to be affected by the hydrolysis degree.^[^ ^1^, ^2^ ^]^ Interestingly, due to the higher availability of the functional side chain (group R) of peptides with lower molecular weight, they may have higher antioxidant activity than peptides with high molecular weight.^[^ ^1^, ^2^ ^]^

#### 2.4.2 Iron Chelating Activity

This test has been conducted by chelation of metal ions, since iron and copper transitions generate reactive oxygen species, such as hydroxyl radicals and superoxide radicals, which lead to unsaturated lipid oxidation.^[^ ^23^ ^]^ The results of the iron‐chelating activity of FPHs in the concentration of 1, 2, 3, and 4 mg mL^−1^ are depicted in Figure 2b. The FPHs showed a dose‐dependent iron chelating activity varying from 30–55% depending on the source of FPH and its dose. FPHs from head and bone showed significantly (*P* < 0.05) higher iron chelating activity than FPH from skin except at a concentration of 3 mg mL^−1^ (*P* > 0.05). Also, in all the evaluated concentrations there were no significant differences between FPH‐head and FPH‐bone (*P* < 0.05). A higher peptide bond cleavage is more likely to explain the increase in metal chelating activity with increasing DH, according to Nikoo et al.^[^ ^23^ ^]^ Thus, low molecular weight peptides can be more strongly charged (carboxyl groups) and have higher mass‐to‐charge ratios, which enables them to form more efficient complexes with metal ions. Senadheera et al.^[^ ^24^ ^]^ reported that peptides can form complexes with transition metal ions to retard the oxidative process after evaluating the antioxidant properties of protein hydrolysates from sea cucumbers (*Cucumaria frondosa*). Based on these results, recovered FPHs could potentially function as antioxidants.

#### 2.4.3 ABTS Radical Scavenging Activity

In the ABTS radical assay, protein hydrolysates from a variety of sources are assessed for their antioxidant activity by reducing the blue/green color of the ABTS radical.^[^ ^17^, ^22^ ^]^ It can also be used to determine lipophilic as well as hydrophilic molecules since the method is based on electron transfer and hydrogen atom transfer mechanisms.^[^ ^24^ ^]^ The results of ABTS scavenging activity of the recovered FPHs are depicted in Figure 2c. Results showed that all the recovered FPHs, regardless of their source, were able to scavenge ABTS radicals well (*P* < 0.05), and at a concentration of 4 mg mL^−1^, were able to eliminate about 70 percent of the radicals. Interestingly, FPH‐head sample exhibited the highest amounts of ABTS scavenging abilities relative to other FPH samples, as in other antioxidant tests. Today, it is well known that several factors such as amino acid composition and sequence, the molecular weight of created peptides, and the degree of hydrolysis are factors that play a very important role in anti‐radical properties against free radicals such as ABTS.^[^ ^17^, ^22^ ^]^ However, each of these factors may produce different results in different samples. For example, in some studies, the free radical capture capacity was increased by increasing the degree of hydrolysis,^[^ ^25^ ^]^ while in some other studies, the opposite result was obtained.^[^ ^16^ ^]^

### 2.5 ACE‐Inhibitory Activity

**Figure 3:**
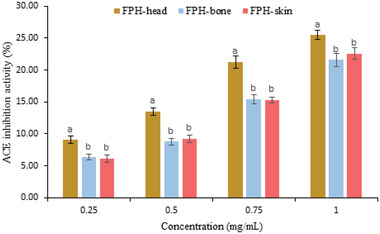
ACE inhibitory activity of fish protein hydrolysates (FPH) recovered from ethanol residue of tuna head, bone, and skin.

The recovered FPH samples after ethanol precipitation were evaluated for ACE inhibitory activity (**Figure** **3**). All the hydrolysates displayed dose‐dependent manner ACE inhibition (*P*<0.05). In all tested concentrations, FPH‐head exhibited the highest ACE inhibitory activity, while FPH‐bone and FPH‐skin exhibited no significant difference (*P* > 0.05). These results could be related to DH of the recovered FPHs as shown in Table 1 that the highest DH was seen in FPH‐head. A similar result was reported by Raghavan and Kristinsson,^[^ ^26^ ^]^ who evaluated the ACE inhibitory activity of protein hydrolysates obtained from tilapia fish by Flavourzyme in two different DH including 7.5% DH and 25% DH, showing that hydrolysates containing 25 percent DH were more effective at inhibiting ACE than hydrolysates containing 7.5 percent DH, suggesting that low MW peptides are more effective at inhibiting ACEs. It has been shown that the ACE inhibitory activity of protein hydrolysates is strongly influenced by hydrophobic amino acids in peptide hydrolysates that inhibit ACE.^[^ ^27^ ^]^ Further, the hydrophobicity of the C‐terminal amino acids and 3D chemical properties significantly affect ACE inhibitory activity, suggesting that hydrophobic amino acids, such as tryptophan, phenylalanine, and tyrosine, have a higher effect on ACE inhibition.^[^ ^26^, ^27^ ^]^ As can be seen in amino acid composition shown in Table 2, FPH‐head contained a high content of phenylalanine and tyrosine. However, Gajanan et al.^[^ ^28^ ^]^ produced protein hydrolysates from *Threadfin bream* by‐products that showed a higher ACE inhibitory activity than our recovered FPHs in the DH rate of 37 to 40%.

### 2.6 Antibacterial Activity

The antibacterial activity of the recovered FPHs is shown in **Table** **3**. The highest inhibition halo size of the recovered FPHs against gram‐positive bacteria including *L. monocytogenes, S. aureus*, and *B. cereus* was seen at FPH‐skin, FPH‐skin, and FPH‐bone with 7.00 ± 0.41, 6.33 ± 0.24, and 3.64 ± 0.24, respectively (*P*<0.05). Also, FPH‐skin, FPH‐head, and FPH‐head showed the highest (*P* < 0.05) antibacterial activity against gram‐negative bacteria *E. coli, S. enterica*, and *S. typhimurium* with the halo zone 5.83 ± 0.47, 5.17 ± 0.47, and 5.00 ± 0.47, respectively. Moreover, by increasing the tested FPH concentration the antibacterial activity of the FPHs was increased. As reported by De Quadros et al.,^[^ ^29^ ^]^ Gram‐negative microorganisms, such as *E. coli*, have an additional structure in their cell wall known as the outer membrane which makes it hard to diffuse antimicrobial compounds into their cells. It has been suggested that the antibacterial activity of the recovered FPHs may have been attributed to the presence of antimicrobial peptides (bacteriocin) produced during the hydrolysis of proteins in the head, bones, and skin.^[^ ^30^ ^]^ FPHs’ antimicrobial activity depends on a variety of factors, including amino acid composition, sequence, molecular weight, structural features, hydrophobicity, hydrophobic moment, charge, and bacterial type.^[^ ^19^ ^]^ In a recent study, it was shown that antimicrobial peptides usually contain between 50 and 100 amino acids, nearly half of which are hydrophobic, with a molecular weight below 10 kDa.^[^ ^30^ ^]^ By forming pores in the cytoplasmic membrane of bacteria, bactriocins can kill the pathogenic bacteria.^[^ ^30^, ^31^ ^]^ Although the peptides’ exact mechanism of antibacterial activity is not yet completely revealed, several peptides could disrupt cellular metabolism and kill the bacteria.^[^ ^32^ ^]^ According to Da Rocha et al.,^[^ ^33^ ^]^ molecular weight could be associated with less aggregate formation, more amino acids being exposed and their charges accumulating, facilitating bacterial binding. Different results have been reported in the study of the effect of degree of hydrolysis on the antibacterial effects of hydrolyzed proteins, which in some studies had a significant effect^[^ ^34^ ^]^ while in others had no significant effect.^[^ ^33^ ^]^ As an example, Wald et al.^[^ ^34^ ^]^ found that rainbow trout by‐product hydrolysates exhibited significant antibacterial activity with increased DH. Therefore, differences in antimicrobial activity between the hydrolysates investigated in this study may be due to differences in amino acid composition. In Table 2, it can be seen that FPH‐skin contains the highest levels of hydrophobic amino acids, which interact with negatively charged bacteria surfaces to penetrate their membrane.

**Table 3:** The antibacterial activity of fish protein hydrolysates (FPH) recovered from ethanol residue of tuna head, bone and skin expressed as inhabitation zone in mm around wells containing 5 or 10 mg mL^−1^ solutions of each FPH

By‐products	FPH‐head		FPH‐bone		FPH‐skin	
Strains	5 mg mL^−1^	10 mg mL^−1^	5 mg mL^−1^	10 mg mL^−1^	5 mg mL^−1^	10 mg mL^−1^
*L. monocytogenes*	3.00 ± 0.41^a^	5.67 ± 0.24 ^B^	2.00± 0.41 ^ab^	5.67± 0.62 ^B^	2.67 ±0.24 ^ab^	7.00± 0.41 ^A^
*S. aureus*	2.17 ± 0.47 ^b^	3.83 ± 0.47 ^B^	2.17± 0.47 ^b^	5.67± 0.24 ^AB^	3.00± 0.41 ^a^	6.33± 0.24 ^A^
*B. cereus*	1.17 ± 0.47 ^b^	3.33 ± 0.85 ^A^	2.00 ± 0.41 ^a^	3.64 ± 0.24 ^A^	1.33 ± 0.24 ^b^	2.83 ± 0.47 ^AB^
*E. coli*	1.67 ± 0.24 ^a^	3.33 ± 0.24 ^C^	2.17± 0.24 ^a^	4.17± 0.47 ^B^	2.33± 0.24 ^a^	5.83± 0.47 ^A^
*S. enterica*	1.83 ± 0.47 ^b^	5.17 ± 0.47 ^A^	3.17± 0.47 ^a^	3.67± 0.62 ^B^	3.17± 0.47 ^a^	4.33± 0.24 ^AB^
*S. typhimurium*	1.83 ± 0.47 ^b^	5.00 ± 0.47 ^A^	2.67 ± 0.24 ^a^	3.83 ± 0.47 ^B^	2.00 ± 0.41 ^ab^	4.33 ± 0.24 ^AB^

Different lowercase letters and capital letters indicate significant differences in 5 mg mL^−1^ and 10 mg mL^−1^ concentrations, respectively, between different FPHs. The values illustrate the means of three replicates ± standard deviations.

### 2.7 Functional Properties

#### 2.7.1 Water Solubility Properties

**Figure 4:**
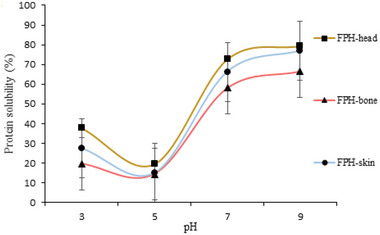
Protein solubility properties of fish protein hydrolysates (FPH) recovered from ethanol residue of tuna head, bone, and skin.

The applications of FPHs in various food applications rely on the availability of high solubility across a wide pH range that can also influence other functional aspects, such as foaming and emulsification activity.^[^ ^28^, ^35^ ^]^ The solubility of recovered FPH samples at different pHs 3, 5, 7, and 9 is depicted in **Figure** **4**. As can be seen in Figure 4, FPH‐head showed the highest solubility in all tested pHs, and also FPH‐skin displayed more solubility than FPH‐bone. Additionally, the results showed that recovered FPHs were soluble at a broad basic pH, which is beneficial for food hydrolysates since it influences emulsification and foaming. Weakly acidic and basic side chain groups of peptides appear to be less soluble at pH 5 and may be explained by protein hydrolysates showing low solubility at their isoelectric points, indicating that pH affects their charge.^[^ ^36^ ^]^ It was interesting to note that FPH‐heads that displayed the highest DH also displayed the highest solubility, indicating that degradation of proteins into smaller peptides results in a marked increase in solubility since the molecular weight of the structure is reduced, the peptide chains unfold, and soluble aggregates are released.^[^ ^25^ ^]^

#### 2.7.2 Foaming Properties

**Figure 5:**
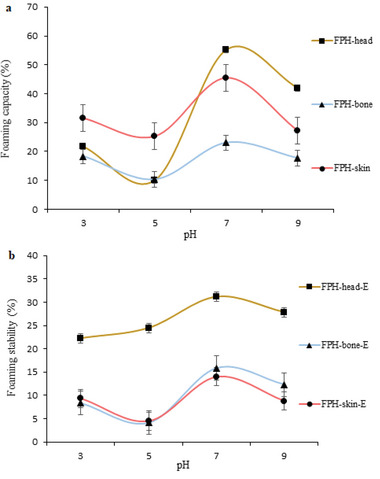
The a) foaming capacity (FC) and b) foaming stability (FS) of fish protein hydrolysates (FPH) recovered from ethanol residue of tuna head, bone, and skin.

The foaming capacity (FC) and foaming stability (FS) of recovered FPH samples are shown in **Figure** **5**a,b. As peptides in the hydrolysates might have varying compositions, sizes, and net charges, the recovered hydrolysates exhibited differences in FC and stability at the tested pH.^[^ ^25^ ^]^ At pH 7, FPH‐head showed the highest FC, and FS among the recovered FPHs. Besides, FC and FS of recovered FPH samples were significantly decreased around acidic pH (*p* < 0.05), and also the highest FC and FS of each recovered FPH were obtained at pH 7. These results are in agreement with Gajanan et al.^[^ ^28^ ^]^ and Halim and Sarbon.^[^ ^37^ ^]^ The researchers noted that peptides with a high molecular weight in protein hydrolysates with low DH have a higher FC and higher stability. Also, a more stable foam could be formed if the hydrolysates contain larger peptides.^[^ ^36^ ^]^ Moreover, in the present study, FC and FS were determined by solubility values at various pHs, and it was clear that solubility values are a significant indicator of their functional properties.^[^ ^35^ ^]^ The foam capacities presented in this study were comparable with the results obtained by Gajanan et al.,^[^ ^28^ ^]^ who used plant proteases to produce protein hydrolysates from fish frame processing by‐products. Moreover, the foaming properties results reported by Mahdabi and Hosseini Shekarabi,^[^ ^38^ ^]^ who evaluated the functional and antioxidant properties of protein hydrolysates obtained from kilka meat, fishmeal, and stickwater were lower than the results obtained in the present work.

#### 2.7.3 Emulsion Properties

**Figure 6:**
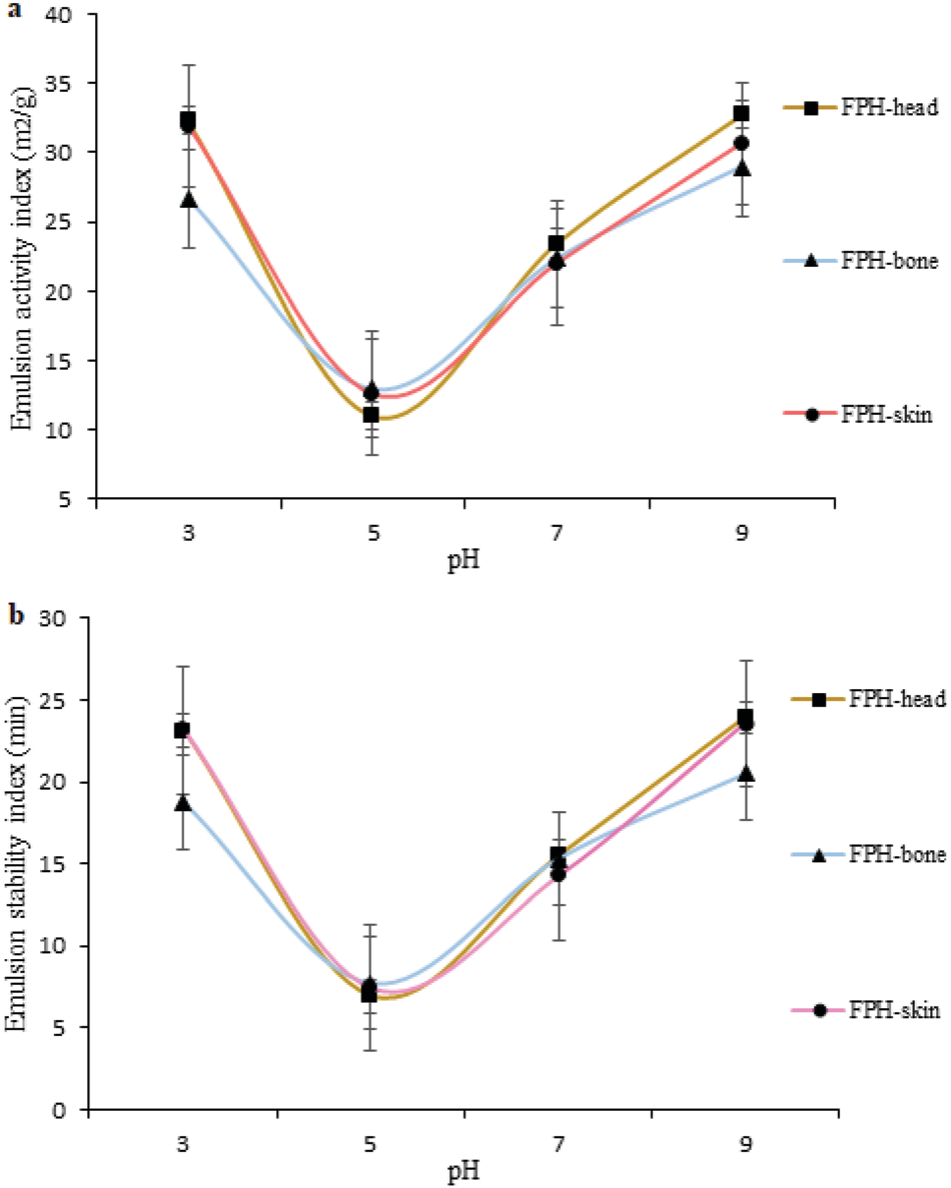
The a) emulsifying activity index (EAI) and b) emulsifying stability index (ESI) of fish protein hydrolysates (FPH) recovered from ethanol residue of tuna head, bone, and skin.

The emulsifying activity index (EAI) and emulsifying stability index (ESI) of FPH‐head, FPH‐bone, and FPH‐skin were investigated and results are shown in **Figure** **6**a,b. In both pH 5 and 7, there was no significant difference (*p* > 0.05), whereas in pH 3 and 9, there was a significant difference (*p* < 0.05) in EAI and ESI. EAI at pH 10 showed the highest values, indicating that polypeptides were unfolding structurally, due to the generation of negative charges that cause repulsion, possibly allowing them to better orient at the interface.^[^ ^39^ ^]^ As a result, these peptides can show increased interaction between hydrophilic and hydrophobic residues, resulting in a major interaction at the oil‐in‐water interface (O: W).^[^ ^28^ ^]^ We found lower EAI and ESI than Pacheco‐Aguilar et al.,^[^ ^39^ ^]^ who evaluated a commercial protease's ability to hydrolyze FPHs obtained from Pacific whiting (*Merluccius productus*) muscle. The study conducted by Gajanan et al.^[^ ^28^ ^]^ examined the properties of protein hydrolysates of frame processing waste produced from plant proteases and concluded that the degree of surface‐active properties could be influenced by DH and the type of enzyme. Additionally, they reported that peptide sizes and sequences are crucial to the emulsifying capacity of peptides. Similarly, a decreasing trend in EAI and ESI at pH 4 and an increasing trend with increasing pH were observed in studies of the antioxidant and functional properties of protein hydrolysates prepared from white shrimp (*Litopenaeus vannamei*). They also found that peptide size and the formation of emulsions were negatively correlated.^[^ ^25^ ^]^

## 3 Conclusions

This study provides useful information about the potential of using the discarded ethanol from the precipitation step in the extraction of polysaccharides from fish by‐products as an innovative source of bioactive peptides. The degree of hydrolysis of the recovered protein hydrolysates was dependent on the type of by‐products and hydrolysates from tuna head displayed the highest degree of hydrolysis. Recovered hydrolysates from the head treatment showed the highest antioxidant activity in all the tests including DPPH and ABTS and Iron chelating activity, and also this sample had the highest ACE‐inhibitory activity compared with hydrolysates from bone and skin. In addition, all the protein hydrolysates displayed antibacterial activity against both Gram‐positive and Gram‐negative bacteria but the antibacterial activity varied between the tested pathogens and samples. Besides, the functional properties including foam and emulsification activity and solubility of the recovered hydrolysates were governed by the type of by‐product used for the production of the hydrolysates and the solubilization pH. Altogether, the results proved that ethanol residue remaining during polysaccharide extraction from skipjack tuna by‐products can be a source for parallel extraction of protein hydrolysates with good bioactivity and functional properties and can be employed as a natural source of antioxidant and antibacterial compounds that could be used in the food and nutraceutical industries.

## 4 Experimental Section

### Materials and Chemicals

The Alcalase enzyme 2.4L (≥2.4 U g^−1^) was procured from Novozymes (Bagsvaerd, Denmark). All chemicals used in gel electrophoresis were bought from Sigma‐Aldrich, along with 1,1‐diphenyl‐2‐picrylhydrazyl (DPPH), ABTS, BSC, bovine serum albumin (BSA), and Folin–Ciocalteu reagent. Additionally, high‐purity and analytical‐grade chemicals and reagents were used in the experiments.

### Sample Collection

By‐products of Skipjack tuna (*Katsuwonus pelamis*) including skin, bones, and head were obtained from a tuna processing company in the Babolsar region of Iran. After packaging of fish by‐products, they were covered with ice at the rate of 1/3 fish by‐product to ice approximately, and transferred to the seafood processing laboratory of Tarbiat Modares University. Until use, samples were washed, packaged, and stored at −24 °C.^[^ ^4^ ^]^

### Enzymatic Hydrolysis and Recovery of Fish Protein Hydrolysate from Ethanol Residue

Polysaccharides of Skipjack tuna were extracted by the enzymatic method and ethanol precipitation according to Jridi et al.^[^ ^4^ ^]^ with some modifications. The tuna by‐products were first homogenized with distilled water and then heated at 90 °C for 20 min to deactivate endogenous enzymes. Then, Alcalase enzyme was added to the suspension at a rate of 1% (w/v enzymes to sample). The pH and temperature of the samples were adjusted to pH 7.5 and 55 °C which was reported as optimum conditions for Alcalase, and hydrolysis continued for 12 h. A 45‐min heating period at 90 °C followed by cooling down to room temperature was then performed on the suspensions. The supernatant was separated from the mixture after centrifugation at 2800 × g for 30 min at 4 °C, and it was then precipitated with absolute ethanol (1:2 v/v) at 4 °C for 12 h. After that, the precipitates were gathered as sulfated polysaccharides. When the precipitating process of sulfated polysaccharide was finished, the ethanol residue from each treatment was collected and evaporated by an evaporator. The condition for ethanol evaporating was 60 °C, 4 h. The time of the process was dependent on the volume of residue ethanol. Then, the obtained concentrated liquid was subjected to freeze‐drying. The concentrated samples were placed in a freezer for 24 h until they were well frozen and then transferred to the freeze dryer. The samples were completely dried and powdered after 48 h. The obtained FPH powder from each by‐product including skin, head, and bone named skin‐FPH, head‐FPH, and bone‐FPH, respectively.

### Degree of Hydrolysis (DH)

DH was determined by using trichloroacetic acid (TCA) of 20%, as previously described by Haslaniza et al.^[^ ^40^ ^]^ In summary, after hydrolysis (12 h), an equal volume of protein hydrolysate was mixed with TCA to make 10% TCA soluble material. The mixed solution of TCA and FPHs was centrifuged at 5 °C (at 6700×g), then the Lowry method was used to calculate protein content. Finally, DH was calculated by the following equation:

(1) $$\begin{array}{c} \text{DH}=\;(\left(\text{Soluble}\;N\;\text{in}\;\text{sample}\;\text{TCH}\;\text{10}\%\left(\text{W/V}\right)\times 100\text{/Total}\;N\;\text{in}\;\text{the}\;\text{sample}\right) \end{array}$$

### Sodium Dodecyl Sulfate Polyacrylamide Gel Electrophoresis (SDS‐PAGE)

SDS‐PAGE was used to fractionate the obtained FPHs as described by Saad et al.^[^ ^41^ ^]^ Initially, 20 g of FPHs were heated for 3 min at 96 degrees Celsius in a mixture containing SDS‐loading buffer. Next, after centrifuging the prepared mixture at 14 000 rpm for 10 min, a 5 µL aliquot was electrophoresed (5 µL of protein/lane). On the stacking gel 10%, the electrophoresis was run at 10 mA toward the positive pole, and on the resolving gel 18%, at 20 mA. After separation, the separated bonds were stabilized in 10% trichloroacetic acid (TCA) for 12 h at ambient temperature. ChemiDoc Gel documentation (BioRad, Hercules, CA, USA) was used to identify separated protein bands and bonds in the range of 10 to 75 kDa were distinguished by a molecular weight marker.

### Amino Acid Analysis

An RP‐HPLC system was used to analyze the amino acid profile of recovered FPHs after hydrolysis in 6 m HCl at 110 °C for 24 h with 1% phenol (v/v). A 2 h precipitation with 10% cold trichloroacetic acid was used to measure the free amino acid profile with the analyzer.^[^ ^42^ ^]^

### ABTS Scavenging Activity

The antioxidant activity of FPHs was assessed as explained by Pezeshk et al.^[^ ^17^ ^]^ with slight modifications. By mixing 7 mm ABTS solution with 2.45 mm potassium persulfate, ABTS radical cation stock was prepared and stored at room temperature for 16 h. Finally, the stock solution was diluted by ethanol to 0.70 at 734 nm. The serial dilution of FPHs was prepared by mixing with water and 50 µL of them were carried to 96‐well microplates and 150 µL of ABTS solution was added to each cell. Then, they were incubated for 20 min. Eventually, the solution absorbance was estimated at 734 nm using an ELISA microplate reader. The ABTS radical scavenging activity was calculated by the following equation:

(2) $$\begin{array}{c} \text{ABTS}\;\text{scavenging}\;\text{activity}\%=\left[Ac-A\text{s/}Ac\right]\times 100 \end{array}$$

Where *A*_c_ was the control absorbance that contained 150 µL of the ABTS solution and 50 µL of ethanol, and *A*_s_ was the sample absorbance solution.

### Iron Chelating Activity

Iron chelating activity of FPHs was determined using the method described by Morales‐Medina et al.^[^ ^43^ ^]^ Summarily, 100 µL of the different concentrations of FPHs, including 1, 2, 3, and 4 mg mL^−1^ added to the mixture that had 450 µL of distilled water and 50 µL of 2 mm FeCl_2_. A five‐minute incubation at room temperature was performed on the mixtures. Next, the mixtures were vigorously shaken and left at room temperature for 10 min after adding 200 µL of ferrozine solution (5 mm). In positive control and control treatments, EDTA and water were used as replacements for the FPHs in the same way. A 562 nm absorbance measurement was conducted, and the chelating activity (%) was determined using the following formula:

(3) $$\begin{array}{c} \text{Metal}\;\text{chelating}\;\text{activity}\left(\%\right)=\left[\left(\text{ODC}+\text{ODB}-\text{ODS}\right)\text{/ODC}\right]\times 100 \end{array}$$

ODC, ODB, and ODS refer to the absorbance's of the control, the blank, and the sample reaction tubes. Triplicate experiments were conducted.

### DPPH Scavenging Activity

Based on the method described by Alboofetileh et al.,^[^ ^44^ ^]^ DPPH scavenging activity was assessed. To prepare the DPPH solution, 25 mL of absolute ethanol was dissolved with 1 mg of DPPH powder. Following that, distilled water was used to prepare FPHs with concentrations ranging from 1, 2, 3, and 4 mg mL^−1^.

Next, 100 µL of FPHs solutions were put into 96‐well microplates. Incubation of the reaction mixture in the dark at room temperature was then conducted for 30 min with 100 µL of DPPH solution added to the FPH sample solution. The absorbance was then measured using an ELISA microplate reader at 515 nm. Based on the following equation, the DPPH radical scavenging activity of samples was calculated:

(4) $$\begin{array}{c} \text{DPPH}\;\text{scavenging}\;\text{activity}\%=\left[Ac-As/Ac\right]\times 100 \end{array}$$

Where *A*_c_ and *A*_s_ defined the control and FPHs sample solution absorbance's.

### ACE Inhibitory Activity Recovered FPHs

According to Roslan et al.^[^ ^45^ ^]^ with a slight modification, ACE inhibition activity was assessed by determining hippuric acid (HA) release from hydrolysis of hippuryl‐histidyl‐leucine (HHL). In the first step, HHL was prepared using potassium phosphate buffer (pH 8.2) that contained 0.3 m sodium chloride. In the same buffer, 60 mU mL^−1^ of ACE was dissolved from rabbit lungs. A mixture of 50 µL of FPH hydrolysates and 50 µL of ACE solution was preincubated for 10 min at 37 °C. Next, 150 µL of HHL solution was added and incubated at 37 °C for 60 min. By adding 250 µL of 1.0 m HCl, the reaction was ended. Following the addition of 400 µL of pyridine, 200 µL of benzene‐sulfonyl‐chloride (BSC) was added. After mixing the solution with a vortex mixer, the solution was cooled on ice. Based on a spectrophotometer measurement at 410 nm, a yellow color was calculated. In order to calculate the percentage of ACE inhibition, the following formula was used:

(5) $$\begin{array}{c} \text{ACE}-\text{inhibitory}\;\text{activity}=\left(A-B/A-C\right) \end{array}$$

In which *A*, *B*, and *C* were the absorbance of the solution without FPH, the solution with the FPH, and the blank solution.

### Bacterial Strains

The antibacterial activity of FPHs was investigated against three Gram‐positive (*Staphylococcus aureus*, *Bacillus cereus*, and *Listeria monocytogenes*) and three Gram‐negative (*Salmonella typhimurium*, *Salmonella enterica, and Escherichia coli*). The microorganisms served for the assay were obtained from Pasteur Institute of Iran: *Listeria monocytogenes* (CMCC 54007), *Staphylococcus aureus* (CMCC 26001), *Bacillus cereus* (ATCC:1247), *Escherichia coli* (O157:H7), *Salmonella enterica* (ATCC 51741), *Salmonella typhimurium* (ATCC 13311).

### Agar Diffusion Method

Briefly, the culture suspensions (200 µL) of bacteria, which their absorbance was 0.08 at 600 nm, were spread on Trypticase soy agar by sterile swap. Next, 50 µL of different FPH solutions (5 and 10 mg mL^−1^) were loaded into cleaned punched wells (6 mm in diameter) in Petri dishes. Thereafter, petri dishes were then placed in an incubator at 37 °C and incubated for 24 h. The antimicrobial activity of the wells was evaluated using the inhibition zone (measured in millimeters) around the wells.^[^ ^17^ ^]^ Triplicates were used for all examinations.

### Emulsifying Properties of FPHs

First, the FPH solutions (30% w/v) were prepared, and after adjusting their pHs in 3, 5, 7, and 9, the emulsion activity index (EAI) and emulsion stability index (ESI) were evaluated.^[^ ^46^ ^]^ To make oil in water emulsion, 9 mL of FPH solutions were blended with 3 mL corn oil. Then, emulsions were homogenized at a speed of 10 000×g for 1 min. After taking 50 µL of the emulsion from the bottom of the beaker, it was mixed with 5 mL of 0.1% sodium dodecyl sulfate (SDS) solution after 10 min. Quickly the mixture absorbance was measured by spectrophotometer at 500 nm. This process again repeats at 10 min to evaluate the emulsion stability index (ESI). Eventually, emulsifying activity index (EAI) and Emulsion stability index (ESI) (min) were calculated by the formulas in follow:

(6) $$\begin{array}{c} \begin{array}{l} \text{Emulsifying}\;\text{activity}\;\text{index}\left(\text{EAI}\right)\left(m^{2}\text{/g}\right)=\\ \;\;\;\left(2\times 2.303\times A500\right)/0.25\times \text{protein}\;\text{weight}\left(g\right) \end{array} \end{array}$$

(7) $$\begin{array}{c} \text{Emulsion}\;\text{stability}\;\text{index}\left(\text{ESI}\right)\left(\min\right)=\left(A0\times \Delta t\right)/\Delta A \end{array}$$

here A500 is the absorbance at 500 nm, ∆A is A0–A10 and ∆t is time at 10 min.

### Foaming Properties FPHs

The foaming properties including FC and stability were assessed by using a method by Halim and Sarbon.^[^ ^37^ ^]^ The pH of the FPHs was adjusted at 3, 5, 7, and 9. Then, about 20 mL of 0.5% solution of the samples were homogenized at a speed of 16 000 rpm for 2 min at room temperature. After this step, immediately, the volume of whipped sample solutions was recorded after 30 s and 3 min. The FC and stability were calculated by the following formula:

(8) $$\begin{array}{c} \text{Foaming}\;\text{capacity}\left(\%\right)=\left[\left(A-B\right)/B\right]\times 100 \end{array}$$

where *A* and *B* were the volume after and before whipping (mL). After reading the total volume of samples, they were allowed to be at room temperature for 3 min. Then, the whole volume of the sample was recorded. The Foam stability (%) was obtained by the following formula:

(9) $$\begin{array}{c} \text{Foam}\;\text{stability}\left(\%\right)=\left[\left(A-B\right)/B\right]\times 100 \end{array}$$

Where *A* and *B* were the volumes of solution after whipping (mL) and before whipping (mL).

### Protein Solubility

The solubility of FPHs was evaluated according to the method reported by Nikoo et al.^[^ ^23^ ^]^ at several pH values (3, 5, 7, and 9). Immediately after centrifuging the hydrolysates at 4000 ×g for 30 min at room temperature, the protein content of the supernatant was determined, and also the total crude protein contents were assessed using the Kjeldahl method (AOAC, 2000) with a Kjeldahl conversion factor of 6.25.^[^ ^47^ ^]^

(10) $$\begin{array}{c} \%\text{Solubility}=\left(\text{Supernatant}\;\text{protein/Total}\;\text{protein}\right)\times 100\% \end{array}$$

### Statistical Analysis

In order to analyze the data, SPSS ver. 22.0, professional edition was used with an ANOVA test. Comparison of the means was conducted using Duncan and differences were considered significant at *p* < 0.05.

## Conflict of Interest

The authors declare no conflict of interest.

## Data Availability

The data that support the findings of this study are available from the corresponding author upon reasonable request.
